# Exploring client perspectives: a qualitative study on how families experience (blended) forensic outpatient systemic therapy for juvenile antisocial behavior

**DOI:** 10.1186/s13034-025-00935-z

**Published:** 2025-07-03

**Authors:** S. Marjolein van Cappellen, Hanneke E. Creemers, Larissa Hoogsteder, Jessica J. Asscher

**Affiliations:** 1 Department of Clinical Child & Family Studies, Utrecht University, P.O. Box 80140, Utrecht, 3584 CS the Netherlands; 2 Faculty of Social and Behavioral Sciences, University of Amsterdam, P.O. Box 15776, Amsterdam, 1011 NG the Netherlands; 3 De Waag, Outpatient Forensic Mental Health Care Center, P.O. Box 1362, Utrecht, 3515 GA the Netherlands

**Keywords:** Forensic outpatient systemic therapy (FAST), Antisocial behavior, Qualitative research, Intervention evaluation, Blended care

## Abstract

**Background:**

Interventions aimed at juveniles exhibiting challenging antisocial behavior often face motivation issues, high drop-out rates, and difficulties in achieving substantial and long-lasting effects. Gaining insight into how families experience these interventions may be a crucial step in obtaining more understanding of what does and does not work for clients in forensic youth care. The current study investigated the experiences of juveniles and caregivers with Forensic Outpatient Systemic Therapy (FAST), an intensive intervention aiming to reduce juvenile antisocial behavior. The study examined how juveniles and caregivers evaluated FAST in terms of the process of setting treatment goals, helpful components, points of improvement, their therapist, and a blended version of FAST (FASTb) that is partially offered online.

**Methods:**

Semi-structured interviews were conducted after treatment termination with 24 participants from 16 families (9 juveniles, 15 caregivers) who participated in a randomized controlled trial or quasi-experimental study on the effectiveness of FAST. A purposive sampling method was used to yield a diverse sample and varied experiences. Thematic analysis was performed in three phases.

**Results:**

Treatment goals were generally set collaboratively and agreement was often achieved, but juveniles were not always involved in setting the treatment goals or in treatment in general. Evaluations of FAST’s success were varied but generally positive. Most helpful treatment components were conversations with the therapist and, to caregivers, program specific components. Although most participants evaluated their therapist positively, some reported the need for more responsivity. FASTb increased treatment accessibility for some caregivers, but most participants preferred face-to-face appointments.

**Conclusions:**

This study provides insight into how FAST is perceived and factors influencing engagement, yielding several clinical implications for systemic treatment in forensic youth care. First, building a strong therapeutic alliance is important. Juveniles benefit from a kind, activity-based approach, whereas caregivers value therapist empathy. Given the severity of antisocial behavior in FAST’s target group, initial safety interventions may be necessary before being able to invest in the therapeutic alliance. Second, therapists should persist in engaging juveniles, for instance, by incorporating (physical) activities. Third, blended care may improve accessibility. Therefore, it is important to discuss its potential with families throughout treatment.

## Background

Interventions aimed at juveniles exhibiting challenging antisocial behavior, such as (domestic) violence, aggression, and delinquent behavior [1], often face difficulties in achieving substantial and long-lasting effects [2, 3], especially in terms of recidivism [4, 5]. Possible explanations are the often mandated referral, as well as characteristics of the target group, making juveniles with antisocial behavior typically less inclined to be engaged in or motivated for treatment [6–9]. In order to increase the likelihood of treatment success, it may be crucial to identify the wishes and needs of those subject to treatment, as gaining insight into client experiences may be an essential step in obtaining a greater understanding of why certain interventions work or do not work [10, 11].

Yet, most intervention research does not focus on the perspectives of clients on the treatment process [11, 12]. Qualitative research can bridge this gap by yielding knowledge on clients’ subjective experiences, opinions, and motivations [12–14]. Moreover, it can provide insight into contextual factors that facilitate or hinder intervention success as well as specific individual needs and resources of a target group [12], which may be crucial for preventing treatment drop-out and for eventual treatment effectiveness.

Therefore, the current study examined how juveniles and caregivers evaluate Forensic Outpatient Systemic Therapy (Forensische Ambulante Systeem Therapie; FAST) [15]. FAST is an intensive, outreaching intervention targeting juveniles showing severe antisocial behavior and their families, who generally have disturbed family relationships and face a multitude of (complex) problems. It aims primarily to reduce juvenile antisocial and/or delinquent behavior, prevent out of home placement, and prevent or decrease recidivism (risk). FAST is based on the social-ecological developmental model of problem behavior [16, 17], according to which both individual and systemic risk factors play a role. Therefore, in line with the Risk-Needs-Responsivity (RNR) model for effectively intervening in antisocial behavior by Bonta and Andrews [18], FAST addresses both individual [19–21] and systemic risk factors that play a role in the development and continuation of antisocial behavior [22–25]. Depending on the needs of the client system, FAST combines systemic interventions and individual treatment, using components originating from system therapy, cognitive behavioral therapy (CBT) with specific attention to aggression regulation therapy, and non-violent resistance [15]. There is special attention for the development of motivation and treatment alliance. For instance, therapists aim to set treatment goals collaboratively with the juvenile and caregiver(s), although this can be a challenge when families exhibit initial resistance or reluctance to engage with goals that address dynamic criminogenic risk factors.

To potentially improve the accessibility and adaptability of FAST, a blended version of FAST (FASTb) was developed, prompted by the worldwide COVID-19 pandemic. FASTb is identical to regular FAST (FASTr) in its content but combines face-to-face treatment with a minimum average of 50% online direct treatment time over the duration of the intervention, such as (video) phone calls. In contrast, FASTr consists of a minimum of 90% face-to-face direct treatment time, mainly through home visits. Offering FAST in a blended form potentially increases its ability to adhere to the needs and responsivity principles of the RNR model [26], as it allows clients to meet the therapist while not being at home and could better suit the learning styles of juveniles. In addition, it simultaneously allows therapists to see more clients in less time. Although blended interventions have several benefits over sole face-to-face treatment [27–29] and therapists view blended interventions as having the potential to improve treatment quality in forensic mental health care, little is known about how clients experience this newer form of treatment in this field [30].

In the context of outpatient forensic systemic youth care, a small number of qualitative studies have investigated the experiences of juveniles and caregivers, mainly with Multisystemic Therapy (MST) [31–33]. FAST shares several similarities with Multisystemic Therapy (MST) and Multidimensional Family Therapy (MDFT) [3, 34], as all three adopt a multimodal approach to treating adolescents with persistent externalizing behavior problems. Each intervention aims to prevent out-of-home placement and to reduce behavioral problems and the risk of recidivism. A key distinction of FAST, however, lies in its integration of individual treatment components. Whereas MST does not include additional individual therapies, FAST is explicitly designed to be combined with individualized interventions that address relevant intrapersonal factors [15, 35]. For example, FAST may be supplemented with aggression regulation training or trauma-focused therapy to meet the adolescent’s specific needs. Although MDFT incorporates individual elements to a limited extent, it primarily focuses on problematic substance use and is generally less outreach-oriented than FAST. With regard to the previous qualitative studies on MST, the study of Tighe et al. [33] reported that although all families experienced some level of improvement, most caregivers noted that not all treatment goals were achieved, and that juveniles were less engaged in treatment compared to their caregivers. Pertaining to facilitators, a recurring theme in these studies was the perceived importance of the therapeutic alliance, i.e., a bond between therapist and client comprising agreement on goals and tasks and bond [36], for initiating and sustaining change [31–33]. However, the generalizability of these results is limited, as the studies predominantly or solely included families that completed treatment, mainly included boys and mothers, or were conducted in a single city [31–33]. In particular, the sole inclusion of families that completed treatment impedes generalizability, as it may positively bias study outcomes [33] and provides limited insights into the factors that hinder positive outcomes [32].

In sum, knowledge on subjective client experiences of treatment success and key factors associated with treatment success is scarce, especially for families that did not complete intervention. Given the hard-to-reach and hard-to-motivate target group of outpatient forensic systemic youth care, enhancing our understanding of how clients experience FAST and its blended version and what factors they experience as crucial for successful treatment may be crucial for preventing treatment drop-out and for increasing treatment motivation and effectiveness.

## The current study

The current study aimed to examine how families experience FAST and the adapted blended version (FASTb), a perspective often overlooked in effectiveness research within forensic youth care. Therefore, it aimed to include a diverse sample of families. Considering the systemic nature of the intervention and the disturbed family relationships that are often present between juveniles and caregivers referred to FAST [15], the current study included the perspectives of both juveniles (boys and girls) and caregivers (mothers and fathers). In addition, families from different treatment locations who did and did not complete the intervention, and families who received FASTr and FASTb were included.

Informed by key elements of FAST [15] and previous qualitative research on client evaluations of MST [33], the current study investigated how juveniles and caregivers evaluate the process of collaboratively setting treatment goals, what components of FAST they perceive as helpful or as points of improvement, and how they evaluate their therapist. In addition, the study explored client evaluations of FASTb, investigating perceived advantages, disadvantages, and points of improvement of the blended approach. By employing a qualitative approach, the study offers an understanding of the factors juveniles and caregivers consider important in FAST, and provides insights into potential improvements for the intervention.

### Methods

#### Study design and sample

The present qualitative study is part of a larger research project aiming to determine the effectiveness of FAST, including a randomized controlled trial (registered at ClinicalTrials.gov, registration number NCT05606978) [37] and a quasi-experimental study. Juveniles and caregivers from both studies were invited to participate in an interview after termination of FAST using a purposive sampling method, aiming to yield a diverse sample and varied information [38]. Therefore, participants were selected based on treatment completion, legal state of referral (as a judicial measure or not), treatment condition, gender, treatment site, age of the juvenile, and therapist. Written informed consent was obtained from juveniles and caregivers for their own participation, and from caregiver(s)/legal representative(s) for juveniles younger than 16 years.

An a priori estimate of the sample size was made to guide the sampling plan [38], whereas the final sample size was determined during data analysis based on data saturation [10, 38, 39]. Phenomenological studies are estimated to require approximately 10 interviews to reach data saturation [38]. However, given that the current study included interviews with both juveniles and caregivers, families who received either FASTb or FASTr, and families who did or did not complete treatment, the estimated needed sample size was *n* = 32 (*n* = 8 juveniles and *n* = 8 caregivers per treatment condition). Data saturation, often defined as the point at which no new analytical information can be discovered [10, 38], was determined to be sufficient when a wide range of experiences from a diverse sample was collected and when themes were recurring across interviews [10, 39, 40].

After 24 interviews were conducted, sufficient saturation was achieved. The participants included *N* = 9 juveniles and *N* = 15 caregivers from 16 families who provided informed consent. Juveniles and/or caregivers from 11 families declined participation (response rate: 59.3%) for various reasons, including not wanting to look back on the treatment, time constraints, or lack of response.

Characteristics of the sample are provided in Table 1. Participating juveniles included seven boys (77.8%) and two girls aged 13 to 16 years (*M* = 14.2, *SD* = 0.97), and participating caregivers included five fathers and 10 mothers. The average recidivism risk of the juveniles based on the RAF GGZ Youth [41] was medium-high to high (*M* = 3.3, *SD* = 1.0). The average treatment duration was 6.6 months (*SD* = 2.5), and the average duration between the termination of FAST and the interview was 2.3 months (*SD* = 2.6). Participants had received FAST from five different treatment sites and from 11 different therapists. Due to purposive sampling, treatment condition was evenly distributed among the participating families, with 12 interviews from eight families for both conditions (FASTr: *N* = 5 juveniles, 7 caregivers; FASTb: *N* = 4 juveniles, 8 caregivers). For both treatment conditions, the percentage of online direct treatment time was determined using therapist report after treatment termination, or when not available, the therapist was asked to make an estimate. When the therapist could no longer be contacted or was unable to provide an estimate, an average was calculated based on the available reports (e.g., monthly caregiver report, case file analysis, supervisor estimate). The majority of families in the FASTb condition received less than the intended 50% of direct treatment time online. Despite, they received substantially more direct treatment time online (*M* = 38.0%, *SD* = 14.6%) than did the families in the FASTr condition (*M* = 8.8%, *SD* = 4.7%). Five families (31.3% of the sample) dropped out of FAST, three of which were based on a mutual decision between family and therapist because there was insufficient motivation, more health care was indicated, and/or caregivers had insufficient caregiving capacity. This could, for example, be related to caregivers’ own psychiatric issues or mild intellectual disabilities. The other two families dropped out because one or more family members terminated the treatment. On average, juveniles from drop-out families were one year older than those from completer families (drop-out: *M* = 15.4, *SD* = 1.14; completer: *M* = 14.4, *SD* = 1.43; *t*(14) = -1.42, *p* =.178, *d* = -0.76) and had a slightly higher recidivism risk score (drop-out: *M* = 3.8, *SD* = 0.45; completer: *M* = 3.1, *SD* = 1.14; *t*(14) = -1.33, *p* =.205, *d* = -0.72). Gender distribution was similar (drop-out: 80.0% boys; completer: 81.8% boys).

**Table 1:** Characteristics of the study sample

Family (condition ^a^)	Participant ^b^, gender	Age (years), gender juvenile	Referral behavior (referral as judicial measure)	Highest domain of recidivism risk (score ^c^)	Primary diagnosis ^d^ (comorbidity)	Treatment duration (months)	Online treatment (%)	Treatment completion	Months between treatment termination and interview
1 (r)	C ♀	13 ♀	Aggression (no)	Violence (3)	OSDICD (yes)	6	5	Yes	4
2 (r)	J ♂ C ♂	13 ♂	Violence (no)	Violence, property (2)	ODD (no)	8	10	Yes	2
3 (b)	J ♂ C ♀	15 ♂	Truancy (no)	Other (1)	ADHD (no)	7	10	Yes	1
4 (r)	J ♂	14 ♂	Domestic violence (no)	Domestic violence (3)	ODD (yes)	10	2	Yes	1
5 (r)	C ♂	17 ♂	Violence (yes)	Domestic violence (4)	OSDICD (yes)	2.5	8	Drop-out (unilateral)	6
6 (r)	C ♀	15 ♂	Property + violence (no)	Violence, property (4)	OSDICD (yes)	8.5	15	Yes	1
7 (b)	C ♀	15 ♂	Violence (yes)	Violence (4)	CD (yes)	3	32	Drop-out (mutual)	1
8 (b)	C ♀	14 ♂	Property, violence, substance use (yes)	Violence (5)	OSDICD (yes)	9	55	Yes	1
9 (b)	C ♀	15 ♂	Domestic violence (no)	Violence, domestic violence, property (3)	UDICD (yes)	2	42	Drop-out (unilateral)	10
10 (b)	J ♂ C ♂	15 ♂	Domestic violence (no)	Domestic violence (2)	UDICD (yes)	9	35	Yes	0.5
11 (r)	J ♂ C ♂ C ♀	16 ♂	Property (no)	Property (4)	OSDICD (no)	4	5	Drop-out (mutual)	0.5
12 (r)	J ♂ C ♀	14 ♂	Property + violence (yes)	Domestic violence (4)	CD (no)	8	15	Yes	4
13 (b)	C ♀	18 ♂	Behavioral problems (no)	Property (4)	CD (yes)	9	50	Yes	0
14 (r)	J ♀	13 ♀	Violence (no)	Violence, domestic violence, other (3)	OSDICD (yes)	7	10	Yes	0.5
15 (b)	J ♂ C ♀	14 ♂	Behavioral problems (no)	Violence (3)	ODD (yes)	7.5	30	Yes	2.5
16 (b)	J ♀ C ♂	14 ♀	Violence (no)	Violence (4)	ODD (yes)	5.5	50	Drop-out (mutual)	1

^a^ b = FAST-blended, r = FAST-regular. ^b^ J = juvenile, C = caregiver. ^c^ 1 = low-medium, 2 = medium, 3 = medium-high, 4 = high, 5 = very high. ^d^ Primary DSM-5 diagnosis [64] of the juvenile. OSDICD = other specified disruptive, impulse-control and conduct disorder, ODD = oppositional defiant disorder, ADHD = attention-deficit/hyperactivity disorder, CD = conduct disorder, UDICD = unspecified disruptive, impulse-control and conduct disorder

## Procedure

This study was approved by the independent Ethics Review Board of the Faculty of Social & Behavioural Sciences of Utrecht University (23–0266). The data were collected between August 2023 and May 2024. Participants were interviewed individually by the first author. The data collection was adjusted as much as possible to accommodate the needs and preferences of the participant. Participants could choose where the interview took place (home visit or video call) and whether the caregiver was present during the interview with the juvenile. Participants received financial compensation of 15 euros for their participation in the interview, although some participants refused it. The average duration of the interviews was 40 min (range: 17–82 min). All the interviews were audiotaped and transcribed verbatim using Amberscript [42] and subsequently deleted. Given that the accuracy of the transcription of Amberscript is between 85% and 90%, the transcripts were checked and corrected by a student assistant. Transcription and data analysis were conducted in Dutch. Quotes were translated into English by the first author.

## Interview

Topic lists for the semi-structured interviews were based on the FAST treatment checklist, an interim evaluation tool to assess the extent to which the potentially effective components of the FAST program are being used [15], and were developed around the four research questions (see Table 2). The national FAST-supervisor and three therapists appointed responsible for treatment integrity of different participating therapist teams provided feedback on the topic lists. After each interview, participants were asked whether, in their opinion, specific questions should be added, altered, or deleted. All participants indicated that the topic list did not need alterations.

The interview questions were formulated as openly as possible to elicit genuine opinions rather than guided responses. If necessary, questions were adapted to suit the participant. Interview questions regarding FASTb were only asked to participants who had received FASTb. Additional interview questions regarding treatment motivation were included in the interview, but are described in a separate article.

**Table 2:** Interview questions per research question, translated from Dutch

Research question	Interview questions
1: How do clients evaluate the process of setting treatment goals?	How did you determine the treatment goals? Who were involved?
2: How do clients evaluate FAST, what do they perceive as helpful components and points of improvement?	To what extent has FAST helped you? Do you think your treatment goals were achieved? What helped you the most? Why? What helped you the least or not at all? Why? Did you miss anything in the treatment? What? What could be improved?
3: How do clients evaluate their FAST therapist?	What did you think of your FAST therapist? What did your therapist do well? What could they improve?
4: How do clients evaluate FASTb?	What did you think of blended FAST? What were the advantages and disadvantages of blended treatment? What could be improved in FAST-blended?

### Intervention

Clients are referred to FAST by the juvenile justice system as a judicial measure or by mental healthcare facilities, school care coordinators, or general practitioners. FAST is an intensive intervention: Each family has a minimum of two appointments per week with their therapist for a period of five to nine months [15], depending on the goals of the client (system), followed by a period of aftercare (see van Cappellen et al. [37] for more information on FAST). FAST distinguishes itself from other systemic interventions for this target group by prioritizing adherence to the RNR principles outlined by Bonta and Andrews [26]. These principles dictate that interventions should be tailored to the risk level of the individual and should target the dynamic criminogenic needs of the client (system) while remaining responsive to their abilities. Within FAST, treatment duration and intensity can be adjusted as needed, and both dynamic protective and risk factors within the client’s broader context are addressed. FAST can be integrated with other treatments to target individual risk factors effectively. Uncontrolled pre-posttest studies investigating the effectiveness of FAST suggested it is effective in decreasing recidivism risk, reducing emotional/personal problems, and improving family functioning [43, 44].

At intervention start, therapists determine recidivism risk, treatment motivation, and safety of the juvenile, caregiver(s), and therapists. FAST targets various treatment goals aimed at reducing problematic behavior. The specific treatment goals for each family are collaboratively determined using a problem behavior Analysis Circle, which also considers the strengths of the system. For each goal, the Analysis Circle is used to identify the most appropriate interventions to achieve the desired treatment outcomes. Treatment goals are jointly evaluated biweekly, and treatment extension is evaluated every two months. Treatment integrity, indicating whether treatment is delivered according to the method and treatment manuals, is closely monitored within FAST, as it can affect treatment results [45, 46]. The FAST team is supervised by a therapist that is responsible for treatment integrity and the national FAST-supervisor, each family is discussed in a multidisciplinary meeting at least every two weeks, and weekly intervision sessions are conducted. If the therapist is a novice, additional supervisory meetings are arranged as needed.

### Data analysis

The transcribed interviews were entered into NVIVO 14 [47] for data analysis. The transcripts were coded in three phases, following Boeije and Bleijenbergh [40]: open coding, thematic coding, and selective coding. First, each transcript was openly coded by administering codes that closely reflected the fragments. Three transcripts were openly coded by the first and second authors, compared and discussed until consensus on the codes was reached. The other transcripts were coded by the first author, ensuring consensus by weekly meetings with the second author. Second, during the thematic coding phase, the codes were clustered per research question, adjusted, and themes were distinguished. For each thematic code, all subcodes were reread to determine whether they reflected the thematic code and, if necessary, moved to a different or new theme. This resulted in the final themes for each research question, which were translated into visual code trees. During this phase, the first and second authors met weekly to discuss doubts and draft code trees. Third, during the selective coding phase, it was analyzed whether specific themes were present or absent based on specific conditions. It was investigated whether there were differences in themes in the interviews of juveniles or caregivers and in the interviews of families that had received FASTb or FASTr, that had completed or dropped out of FAST, and that were, or were not, referred to FAST as a judicial measure. Finally, all final code trees were reviewed by all authors to ensure consensus, and results were described per topic. In the Results section, only themes resulting from three or more participants are presented. Two exceptions are results from juveniles and results regarding FASTb. As these originate from smaller subsamples, themes resulting from two or more participants are presented.

## Results

### Research question 1: how do clients evaluate the process of setting treatment goals?

**Fig. 1:**
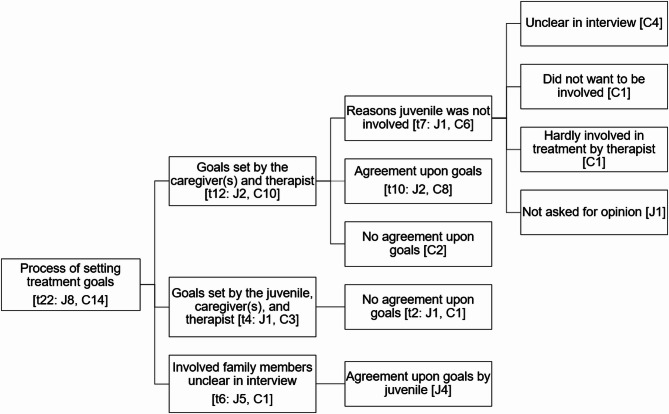
Code Tree Research Question 1: How Do Clients Evaluate the Process of Setting Treatment Goals? Note: The numbers in square brackets indicate the number of participants reporting a theme: t = total number of participants, J = number of juveniles, C = number of caregivers

Three themes emerged from the data regarding the process of setting treatment goals: involvement of family members, reasons juveniles were not involved in treatment goal setting, and agreement (see Fig. 1).

### Involvement of family members

According to half of the participants (two juveniles, 10 caregivers), treatment goals were set collaboratively by the caregiver(s) and therapist, without the involvement of the juvenile. Four participants (one juvenile, three caregivers) described that the juvenile was involved in treatment goal setting as well. In six interviews, it remained unclear who was involved.

### Reasons juveniles were not involved in treatment goal setting

A few participants elaborated on the process of setting treatment goals. For example, one juvenile mentioned that in the conversation on treatment goal setting, only his mother was asked what she wanted to change. One father explained that his son did not want to be involved in the treatment goal setting because he did not care about the treatment, whereas another father shared that his daughter was hardly involved in the treatment by the therapist in general.

### Agreement

Most participants (58.3%, six juveniles, eight caregivers) voiced that there was agreement upon the treatment goals between the family and therapist. For example, several participants mentioned that the goals were in line with the problems they were experiencing as the most severe, and many caregivers experienced that treatment goals were set based on their presenting issues or an analysis of the experienced problems. Some experienced that the treatment goals were solely based on their own input, whereas others experienced that their therapist also added treatment goals to a greater or lesser extent.

Some participants (one juvenile, three caregivers) mentioned that some treatment goals were not agreed upon by all family members. For example, one caregiver described that her son protested against the treatment goal of him going to school, as he did not want to return to school. One father described a lack of agreement upon the treatment goals due to defective problem analysis: “*I cannot judge that the therapy is not good*,* but I* can *judge that the problem was not identified*,* eh*,* well enough. Or at least*,* the* actual *problem.”* A notable finding was that the majority of families that dropped out of FAST reported disagreement on treatment goals (three out of four families that reported on this). Moreover, participants who experienced a lack of agreement on treatment goals evaluated the success of FAST more negatively.

### Research question 2: how do clients evaluate FAST, what do they perceive as helpful components and points of improvement?

On average, participants graded the FAST program as 6.8 (*SD* = 2.0, range: 2.0–10.0). With regards to the perceived treatment success of FAST, participants indicated that FAST helped them very well (two juveniles, six caregivers), well (one juvenile, three caregivers), a little (two juveniles, one caregiver), not really (two juveniles) or not at all (two juveniles, four caregivers). In terms of treatment goal achievement, most participants (five juveniles, six caregivers) believed that their treatment goals were achieved, although two juveniles did not attribute this to FAST. Six participants (three juveniles, three caregivers) believed that their goals were partially achieved, and five participants (one juvenile, four caregivers) believed that none of their goals were achieved. Notably, in this context as well, participants from families who dropped out of FAST evaluated its success more negatively: Six participants (75%) reported not having achieved any goal and/or reported that FAST did not help them at all. There seemed to be no differences in the evaluations of FAST’s success between families receiving FASTb or FASTr and between families with or without referral to FAST as a judicial measure.

Several participants (one juvenile, three caregivers) mentioned that an unachieved goal to them was a diagnostic trajectory to obtain a diagnosis for the juvenile. These caregivers stated that their wish for a diagnostic trajectory had been expressed by them but that it had not been (fully) addressed by the therapist. For example, a mother said: “*So*,* well*,* they would look at that. Eh*,* but every time we asked back on that it was still looked at*,* and nothing was ever done about it.*” Furthermore, four caregivers described that it was difficult for them to combine the intensity of FAST with their jobs. FAST required them to take time off of work due to frequent appointments during office hours, which was challenging for them.

### Most helpful components

**Fig. 2:**
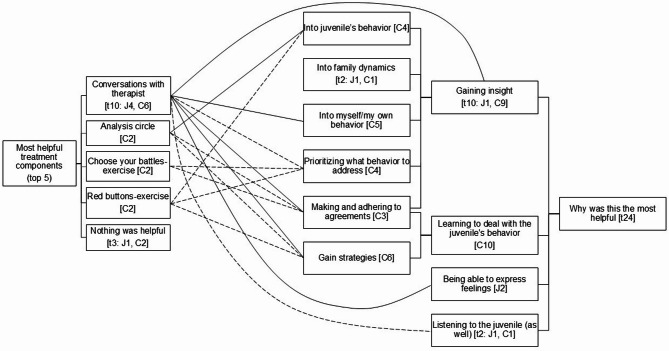
Code Trees Most Helpful Treatment Components and Why Was This the Most Helpful. Note: The numbers in square brackets indicate the number of participants reporting a theme: t = total number of participants, J = number of juveniles, C = number of caregivers. The solid lines connecting the left code tree with the right code tree indicate reasons described more than once, and the dotted lines indicate reasons described once

Apart from a few participants who stated that nothing was helpful, two main themes emerged from the data: conversations with the therapist and program specific components (see Fig. 2). Participants gave several reasons how these components helped them the most, which could be categorized as to gain insight (one juvenile, nine caregivers), to learn to deal with the child’s behavior (10 caregivers), and to be able to express feelings (two juveniles).

**Conversations With the Therapist.** Many participants (41.7%, four juveniles, six caregivers) indicated that the conversations they had with their therapist were most helpful to them. Four caregivers argued that the conversations helped them the most because they taught them to deal with the juvenile’s behavior and to gain insight into themselves, such as their own behavior or interaction style. For example, one father described: “*The most difficult thing is that as a parent*,* you have to reflect very strongly on yourself. But that is the most valuable thing*,* because that is probably quite*,* yes*,* the key to*,* eh*,* changing your child’s behavior.*” Two juveniles indicated that the conversations with their therapist helped them express their feelings, for example: “*Just talking a lot and just sharing feelings (…) At first*,* I also really did not feel like talking at all*,* and I just rather did not want any help at all. But eh yes*,* when things are going badly*,* talking is the best solution after all.*”

**Program Specific Components.** Eight caregivers mentioned specific program components of FAST as being most helpful, with three components mentioned by two caregivers each, namely Analysis Circles (see Methods), Choose your Battles, and Red Buttons. The purpose of the Choose your Battles-exercise is to concentrate on problem areas where successes can be attained while ensuring that conflicts do not escalate by emphasizing every minor issue. The purpose of the Red Buttons-exercise is to help caregivers recognize which behaviors of their child trigger strong emotional responses in them, as if a ‘red button’ is being pressed. Caregivers identify their top five triggers and learn strategies for responding to them in a way that aligns with their caregiving role and effectively addresses the child’s needs. Caregivers felt that these exercises helped them the most because they helped them gain insight into their child’s behavior, make clear agreements and adhere to them, and gain new strategies to deal with their child’s behavior. For instance, one father described: “*And the red buttons were a thing (…)*,* because he does that a lot to me. He knows exactly what to say to me to*,* eh*,* to get me on the fence. And then [the therapists] explained that and I find it*,* I found it incomprehensible that a child… That the negative attention they ultimately get because of that*,* that they apparently* want *that. So now I let myself be played a little less*,* eh*,* in that respect.*”

### Missed treatment components and points of improvement

In total, 16 participants (six juveniles, 10 caregivers) reflected on whether they missed anything during FAST. Eight participants (four juveniles, four caregivers) mentioned not having missed anything. In addition to the aforementioned participants who expressed that they had missed a diagnostic trajectory, four caregivers mentioned that they had missed therapist responsivity to their wish or request, such as being open to finding the middle ground between views on parenting, involving the juvenile enough in treatment, or following caregivers’ advice on how to involve the juvenile. For example, one mother described FAST had been helpful for her but not for her son, as her sons appointments with his therapist were discontinued after only a few appointments because “*[he] was no longer open to that*,* and they didn’t get any further. (…) That’s the only pity*,* because I had said that beforehand. With [son]*,* you first have to build a bond. And then you can talk to him*,* and everything. And that wasn’t really done. (…) Just invest [i.e.*,* in the bond] first and don’t - bang! - start the treatment right away. Because if someone doesn’t trust you*,* they won’t say anything either.*”

In addition, 16 participants (nine juveniles, seven caregivers) reflected on whether they had any points of improvement. Three main themes emerged from the data. First, five participants (three juveniles, two caregivers) thought that FAST could be improved if the appointments involved less “just” talking and more (physical) activities for juveniles, such as taking a walk outside (three participants), boxing, working out, or playing soccer. According to the participants, talking and sharing are easier for juveniles when they are doing an activity. Second, four participants (two juveniles, two caregivers) mentioned that FAST can be improved if all family members are involved (more). The two juveniles expressed they would have wanted to be more involved in the treatment themselves. One described there could have been more conversations with him, as most were only with his parents. The other juvenile reported being invited to only one appointment by his therapist, even though he was informed that involving him was one of the goals of FAST. His treatment motivation would have been higher if that goal had been pursued: “*Just follow the goals. (…) That if you say something*,* you actually follow through on it. (…) For example*,* here [i.e.*,* in FAST] they say: “conversation with everyone”. And then you [should] really have conversations with everyone. Instead of just with parents.*” Third, three caregivers described that the treatment trajectories of the juvenile and caregiver were too separate. Two of them described that they only had separate appointments and missed appointments with the juvenile and caregiver(s) together: “*It is about that interaction between parent and child*,* and that is then [i.e.*,* in family meetings] also directly observed well.*”

### Research question 3: how do clients evaluate their FAST therapist??

In the evaluation of their FAST therapists, almost all participants mentioned positive experiences and characteristics (eight juveniles, 14 caregivers). Additionally, six participants also mentioned negative experiences with and characteristics of their FAST therapist (three juveniles, three caregivers).

**Positive Experiences and Characteristics.** Three main themes emerged from the data regarding the positive characteristics described on FAST therapists: cooperative relationship, professional skills and knowledge, and positive attitude.

***Cooperative Relationship.*** Many participants (five juveniles, 12 caregivers) experienced a cooperative relationship with their therapist. This relationship was characterized for them by trust (three juveniles, eight caregivers), a connection (two juveniles, six caregivers), and a devoted therapist (three caregivers). For instance, a mother described her immediate connection with her therapists: “*I had*,* from the moment I actually met her*,* I just had a good connection*,* I think (…) [the] first impression [of the therapists] was already like: they feel familiar*,* honest*,* also just sincere*,* just honest right away.*”

***Professional Skills and Knowledge.*** Many participants (five juveniles, 11 caregivers) appreciated the professional skills and knowledge of their therapist. For example, nine participants (four juveniles, five caregivers) stated that their therapist had good conversation skills, the most prominent one being good at listening (two juveniles, two caregivers): “*Just the way the interaction went*,* just the real listening… The real listening*”. In addition, several participants mentioned they found their therapist experienced (one juvenile, two caregivers), knowledgeable (three caregivers), solution oriented (three caregivers), could deal well with the juvenile (one juvenile, two caregivers), and paid attention to differences between family members (one juvenile, two caregivers).

***Positive Attitude.*** Many participants (four juveniles, nine caregivers) felt that their therapist had a positive attitude. Participants described their therapists as being kind or nice (four juveniles), understanding (one juvenile, three caregivers), and empathetic (three caregivers). A caregiver described: “*Yeah*,* he was so full of empathy. And that helped tremendously. In the past*,* I always felt a little bit*,* eh*,* that you’re not doing it right (…) And he was very clear*,* very firm*,* but he showed me how hard I’m trying. So I’m incredibly grateful.*”

**Negative Experiences and Characteristics.** Two main themes emerged from the data regarding the perceived negative characteristics of FAST therapists. Three participants (one juvenile, two caregivers) had experienced trust issues with their therapist. For example, one caregiver described that confidential information was shared with their child without their permission or knowledge. One juvenile described how her therapist kept asking questions about topics she was not ready to talk about: “*The moment you indicate that you don’t like something*,* they [i.e.*,* the therapists] continue anyway. And for many juveniles that is just something that they just cannot*,* that they can’t bear in their head if they already*,* if they already have anger problems. And so they try to provoke something (…) [they] just keep asking questions and if the trust is not there*,* then your motivation is also gone (…) that trust I had in them was just very little.*” The same two caregivers and one other juvenile experienced their therapist as being negative in their communication, such as being too direct or not respectful: “*He was very sharp and very decisive. I thought that was actually*,* in the beginning*,* that was very good*,* because he did get to the point and he knew how to get to you*,* so to speak. (…) But at a certain point*,* he became a bit too*,* too much. (…) And then I was basically laughed at for my vision on parenting*,* so to speak. (…) Well*,* that didn’t go down so well of course. From that moment on*,* eh*,* the entire treatment did not go well*”. Notably, participants from families who dropped out of FAST were more likely to report negative characteristics of or experiences with their therapist (37.5%) compared to those from families who completed FAST (18.8%).

### Points of improvement

Two main themes emerged from the data from 15 participants who reflected on points of improvement for their FAST therapist. Most of these participants (73.3%, three juveniles, eight caregivers) reported not having any points of improvement for their therapist. In contrast, two juveniles indicated that their therapist should communicate more gently toward them: “*Look*,* [therapist] said: “yes you really have to do better quickly*,* otherwise you’ll go out of home” and things like that. And then*,* that’s just*,* just*,* every time*,* not*,* I just didn’t like it*.”

### Research question 4: how do clients evaluate FASTb?

There were no differences in the evaluations of FAST’s success between families that had received FASTb or FASTr. In addition, there was no difference in the drop-out rate between the two conditions (two families dropped out in FASTr, three families dropped out in FASTb).

In total, 12 participants (four juveniles, eight caregivers) had received FASTb and were interviewed on their evaluation of FASTb. Three themes emerged from the data: preference for face-to-face or online treatment appointments, advantages of blended treatment, and disadvantages of blended treatment.

### Preference for face-to-face or online treatment appointments

Eight participants (one juvenile, seven caregivers) who had received FASTb expressed that they had preferred either face-to-face or online treatment appointments within FASTb. The majority of these participants (one juvenile, five caregivers) expressed a preference for the face-to-face appointments within FASTb. Two caregivers expressed a preference for being able to have online appointments in addition to face-to-face appointments. One mother described that it is pleasant to be able to have online appointments if, for example, she is not doing well mentally. Another mother found the alternation between online and face-to-face appointments helpful and found it increased treatment feasibility for her. The reasons given by the caregivers for preferring face-to-face appointments correspond with the experienced disadvantages of FAST-blended, which are described below.

### Advantages of blended treatment

Eight participants described experienced advantages of FASTb. Five caregivers (62.5%) mentioned flexibility, for example, because online appointments take less time and save travel time. Four participants, including one juvenile, experienced convenience as an advantage. For example, one mother mentioned not having to tidy or clean her house for an online appointment, and one juvenile said that during an online appointment, instead of sitting neatly at the table, he can sit on the couch. Furthermore, two caregivers mentioned that it is an advantage of FASTb that the therapist can be contacted in the moment, for example, to discuss an emergency situation.

### Disadvantages of blended treatment

Nine participants described experienced disadvantages of the online appointments. Four caregivers stated that they experienced a lower sense of connection with their therapist during online appointments. For example, they described online appointments as being more superficial. Three caregivers believed that a disadvantage of online appointments is that nonverbal communication is less visible. Additionally, two participants (one caregiver and one juvenile) experienced less focus during or following online or telephone appointments, and two caregivers thought video calling was a disadvantage, as they do not like that in general.

Despite the mentioned disadvantages, all participants noted that they had no points of improvement for FASTb. For example, one mother described: “*That I do not know. I think it really depends on the way a person would like to receive [the treatment]*”.

## Discussion

The aim of the present study was to provide a comprehensive understanding of how juveniles and caregivers experience FAST, a perspective often overlooked in effectiveness research within forensic youth care. Specifically, the study examined how juveniles and caregivers evaluated the process of collaboratively setting treatment goals, which components of FAST they perceived as helpful or in need of improvement, and their therapist. Additionally, it explored client evaluations of FASTb, examining perceived advantages, disadvantages, and areas for improvement in the blended approach.

With regard to the collaborative process of setting treatment goals, caregivers evaluated their involvement in treatment goal setting positively, and most participants experienced agreement upon the treatment goals. The majority of families who dropped out of FAST reported disagreement on treatment goals, and participants who experienced a lack of agreement evaluated the treatment success of FAST more negatively. This finding aligns with meta-analytic evidence linking agreement on treatment goals to better treatment outcomes [48]. The majority of participants described a lack of juvenile involvement in treatment goal setting. This is in contrast with FAST’s aim to involve the juvenile in goal setting [15], as a lack of juvenile involvement in this process has been negatively associated with treatment motivation [49], completion [50] and outcomes in forensic youth care [51]. In sum, the current study shows that treatment goal agreement is important for both treatment completion and outcomes. Moreover, while caregivers are generally satisfied with their own involvement in treatment goal setting, effectively involving juveniles remains a challenge.

Although most participants indicated that FAST had helped them to some extent, evaluations were mixed. This is consistent with previous qualitative research in systemic forensic youth care [33] and is not surprising, as both families that completed and dropped out of FAST were included in the study. Indeed, participants from families who dropped out of FAST evaluated the success of FAST more negatively. Further study is needed to determine whether this reflects a decreased effectiveness of the intervention. There seemed to be no differences in evaluations of FAST’s success between families referred to FAST with or without a judicial measure. This may be attributed to the limited distinction between these two groups within the FAST population: In cases where families are referred to FAST without a judicial measure, the referral is often influenced by external pressures, such as from healthcare or safety institutions, which makes it not entirely voluntary.

Caregivers considered specific components of FAST to be most helpful. In line with the objectives of FAST [15], these components provided them with concrete strategies to deal with their child’s behavior and helped them gain insight into themselves and their family (members). Several points of improvement related to the systemic nature of FAST, as some participants indicated that FAST could be improved by involving all family members (more) in the treatment process and by integrating the treatment trajectories of the juvenile and caregiver(s) more. Specifically, participants emphasized the importance of involving juveniles themselves in treatment, which is in line with previous research [52–54] and is a fundamental component of FAST [15]. This is particularly important, as individual risk factors, in addition to systemic risk factors, play a role – factors that cannot be addressed solely by modifying caregivers’ responses to their child’s behavior. However, involving juveniles in forensic youth care is challenging [6, 8, 33]. Several participants suggested integrating more (physical) activities for juveniles to enhance engagement. The incorporation of experiential techniques may indeed be beneficial, as these techniques are known to enhance treatment effectiveness when combined with CBT for juveniles with persistent externalizing behavioral problems [55]. Additionally, incorporating more (physical) activities may promote the specific responsiveness of the intervention [26] and may align with the learning style of juveniles, thereby increasing treatment effectiveness.

Other findings on treatment components that were helpful, missed, or could be improved overlapped with therapist evaluations, as they related to the therapeutic alliance. Many participants considered conversations with the therapist the most helpful treatment component, and while experiences varied, most participants reported positive perceptions of their therapist and evaluated their FAST therapist favorably. Participants described key elements that are part of the definition of therapeutic alliance [36], including cooperation, professional skills and knowledge, responsiveness, and trust. Interestingly, juveniles and caregivers seemed to define elements of therapeutic alliance differently or to attach importance to different aspects of it: Juveniles mainly described their therapist as being kind or nice, whereas for caregivers, this involved being understanding and empathetic. The present study appears to be consistent with previous research in which the therapeutic alliance is conceptualized differently for youth and adults (e.g [32]). The prominent role of the therapeutic alliance in client perspectives on FAST aligns with findings from previous qualitative studies in forensic youth care [31–33].

Next to positive themes regarding therapeutic alliance, several negative evaluations and experiences were described. Some caregivers mentioned having experienced a lack of therapist responsiveness to their wishes regarding diagnostics or treatment processes, and some participants described having experienced trust issues and unpleasant or negative communication. Participants who dropped out of FAST were more likely to report negative experiences or characteristics of their therapist than participants who completed FAST, which is consistent with (meta-analytic review) studies suggesting a link between weak therapeutic alliance and treatment drop-out within forensic youth care [56, 57]. These results emphasize the importance for therapists to be aware of how their communication is perceived, to ensure not to harm the therapeutic alliance. In addition, the results underscore the crucial role of the therapeutic alliance from a client perspective: Whereas a positive alliance seems to promote perceived treatment success, a negative alliance may be damaging.

It is important to note that treating this target group inherently involves encountering resistance or discontent, as reflected not only in these results but also in the results which indicated that it can be challenging to involve juveniles in treatment goal setting and to reach goal agreement with families. These difficulties can result from the antisocial and psychiatric problems faced by not only the juvenile, but often the caregiver(s) as well [15]. These results reflect the tension between the importance of establishing a therapeutic alliance [58, 59] and increasing treatment motivation [6–8] within FAST, versus the necessity of addressing dynamic criminogenic risk factors [26] and implementing certain (safety) interventions at the outset of treatment [15]. This means that therapists cannot always (immediately) respond to requests or wishes from clients, that clients may resist working on certain goals that are professionally deemed necessary, and that it can be difficult to realize and retain a strong therapeutic alliance. The current study suggests that, when goals diverge, it is important that therapists clarify in a responsive way why a requested goal is not addressed or why a certain goal does have to be worked on. Future research should further investigate how therapists can navigate these often conflicting interests, needs, and wishes. It is important that therapists acquire the necessary skills to address these challenges and are supported by, for instance, case discussions, supervision, and further education to carry out this challenging task.

### Client evaluations of FASTb

The current study aimed to explore how clients experienced the blended version of FAST. Drop-out and evaluations of the success of the intervention did not seem to differ between families that had received FASTr and those that had received FASTb. Despite evaluating FASTb generally positively, most participants expressed a preference for face-to-face treatment appointments, although two caregivers preferred being able to have online appointments in addition to face-to-face appointments, as this increased treatment accessibility. Experienced advantages of FASTb also included increased flexibility and convenience, which aligns with prior research showing that blended treatment improves accessibility [28, 29] and flexibility [29]. While these advantages might increase adherence of FAST to the RNR principles [26] for some families, experienced disadvantages suggest that FASTb may not meet the responsivity principle or could negatively affect therapeutic alliance for others. However, previous research, although outside of forensic youth care, found no differences in therapeutic alliance when comparing blended or online interventions with face-to-face interventions [60–62]. In sum, the current study suggests that therapists evaluate the suitability of blended treatment for each family throughout the intervention, adjusting online session frequency and timing as needed.

#### Study limitations

The current study has several limitations. First, the study yielded limited insight into juveniles’ experiences with blended intervention. Although juveniles were interviewed on their evaluations of FASTb, most juveniles reported that the online appointments were held mainly with their caregiver(s). Second, although the study included a diverse sample, it may not fully represent the families who received FAST. Consequently, the findings may not entirely reflect the overall experiences with FAST or the frequency of these experiences. However, our primary objective was to ensure diversity rather than representativeness, as qualitative research aims to capture a broad spectrum of perspectives [38], including those of participants who did not complete the treatment [11, 63]. Third, although a key strength of the current study lies in its qualitative methodology, due to the absence of treatment outcome data, the findings of this study cannot be interpreted in the light of objective treatment effects.

#### Conclusions and implications for clinical practice

This study explored the experiences of juveniles and caregivers with FAST, yielding several implications that can benefit the implementation of systemic treatment in the context of forensic youth care. First, a focus of therapists on building therapeutic alliances with juveniles and caregivers seems crucial. For juveniles, a focus on applying a positive basic attitude of kindness, gentle communication, and integrating physical activities such as walking seems beneficial. For caregivers, an empathetic, understanding, and respectful approach may strengthen the alliance. This must, however, be considered in the context of the intervention, which targets juveniles that can exhibit severe forms of antisocial behavior. Therefore, it may first be necessary to implement interventions aimed at ensuring the safety of the juvenile and/or others before being able to invest in building a therapeutic alliance. Second, therapists should aim to involve juveniles more in treatment. Although FAST explicitly emphasizes the importance of providing individual treatment for juveniles [15], reaching and engaging these juveniles can be challenging [6–9]. This underscores the importance for therapists to demonstrate persistence in engaging with the youth, exploring various approaches to connect with them, and considering their learning styles, such as incorporating more (physical) activities into treatment sessions. Third, it is plausible that blended care could enhance the accessibility of FAST for some clients. Although the effectiveness of FASTb has yet to be established, discussing the possibility of a blended treatment approach with families throughout intervention could help assess whether it improves accessibility and, consequently, their engagement.

## Data Availability

Due to the sensitive nature of our data, the data of the current study are not publicly available.

## Abbreviations

FAST: Forensic Outpatient Systemic Therapy (Forensische Ambulante Systeem Therapie)
FASTb: Blended Forensic Outpatient Systemic Therapy
FASTr: Regular Forensic Outpatient Systemic Therapy
RNR: Risk-Needs-Responsivity
CBT: Cognitive Behavioral Therapy
MST: Multisystemic Therapy
MDFT: Multidimensional Family Therapy
